# Ethanolic extract of *Origanum syriacum L.* leaves exhibits potent anti-breast cancer potential and robust antioxidant properties

**DOI:** 10.3389/fphar.2022.994025

**Published:** 2022-10-10

**Authors:** Joelle Mesmar, Rola Abdallah, Kamar Hamade, Serine Baydoun, Najlaa Al-Thani, Abdullah Shaito, Marc Maresca, Adnan Badran, Elias Baydoun

**Affiliations:** ^1^ Department of Biology, American University of Beirut, Beirut, Lebanon; ^2^ UMRT INRE 1158 BioEcoAgro, Laboratorie BIOPI, University of Picardie Jules Verne, Amiens, France; ^3^ Breast Imaging Section, Imaging Institute, Cleveland Clinic Foundation, Cleveland, OH, United States; ^4^ Research and Development Department, Barzan Holdings, Doha, Qatar; ^5^ Biomedical Research Center, College of Medicine, and Department of Biomedical Sciences at College of Health Sciences, Qatar University, Doha, Qatar; ^6^ Aix-Marseille University, CNRS, Centrale Marseille, iSm2, Marseille, France; ^7^ Department of Nutrition, University of Petra, Amman, Jordan

**Keywords:** herbal medicine, phytochemical content, breast cancer, metastasis, oxidative stress, reactive oxygen species, ROS, *Origanum syriacum* L

## Abstract

**Background:** Breast cancer (BC) is the second most common cancer overall. In women, BC is the most prevalent cancer and the leading cause of cancer-related mortality. Triple-negative BC (TNBC) is the most aggressive BC, being resistant to hormonal and targeted therapies. Hypothesis/Purpose: The medicinal plant *Origanum syriacum L.* is a shrubby plant rich in bioactive compounds and widely used in traditional medicine to treat various diseases. However, its therapeutic potential against BC remains poorly investigated. In the present study, we screened the phytochemical content of an ethanolic extract of *O. syriacum* (OSEE) and investigated its anticancer effects and possible underlying mechanisms of action against the aggressive and highly metastatic human TNBC cell line MDA-MB-231. Methods: MTT, trans-well migration, and scratch assays were used to assess cell viability, invasion, or migration, respectively. Antioxidant potential was evaluated *in vitro* using the DPPH radical-scavenging assay and levels of reactive oxygen species (ROS) were assessed in cells in culture using DHE staining. Aggregation assays were used to determine cell-cell adhesion. Flow cytometry was used to analyze cell cycle progression. Protein levels of markers of apoptosis (BCL-2, pro-Caspase3, p53), proliferation (p21, Ki67), cell migration, invasion, or adhesion (FAK, E-cadherin), angiogenesis (iNOS), and cell signaling (STAT3, p38) were determined by immunoblotting. A chorioallantoic Membrane (CAM) assay evaluated *in ovo* angiogenesis. Results: We demonstrated that OSEE had potent radical scavenging activity *in vitro* and induced the generation of ROS in MDA-MB-231 cells, especially at higher OSEE concentrations. Non-cytotoxic concentrations of OSEE attenuated cell proliferation and induced G_0_/G_1_ cell cycle arrest, which was associated with phosphorylation of p38 MAPK, an increase in the levels of tumor suppressor protein p21, and a decrease of proliferation marker protein Ki67. Additionally, only higher concentrations of OSEE were able to attenuate inhibition of proliferation induced by the ROS scavenger N-acetyl cysteine (NAC), indicating that the anti-proliferative effects of OSEE could be ROS-dependent. OSEE stimulated apoptosis and its effector Caspase-3 in MDA-MB-231 cells, in correlation with activation of the STAT3/p53 pathway. Furthermore, the extract reduced the migration and invasive properties of MDA-MB-231 cells through the deactivation of focal adhesion kinase (FAK). OSEE also reduced the production of inducible nitric oxide synthase (iNOS) and inhibited *in ovo* angiogenesis. Conclusion: Our findings reveal that OSEE is a rich source of phytochemicals and has robust anti-breast cancer properties that significantly attenuate the malignant phenotype of MD-MB-231 cells, suggesting that *O. syriacum* may not only act as a rich source of potential TNBC therapeutics but may also provide new avenues for the design of novel TNBC drugs.

## 1 Introduction

Cancer is a leading cause of death worldwide, having claimed an estimated 10 million deaths in 2020. Breast cancer (BC) is the most common cause of new cancer cases and the fifth leading-cause of cancer-related deaths (Who, 2021). Moreover, the incidence of BC has increased significantly in recent years to become the world’s most prevalent cancer (Breast Cancer, 2021). Despite significant advancements in treatment regimens and modalities, treatment of most types of breast cancer is still limited to surgery, chemotherapy, and irradiation. Hormone replacement therapy can be used for breast cancer subtypes that are positive for the estrogen receptor (ER) or progesterone receptor (PR), while targeted therapies using antibodies, like trastuzumab, is effective against breast cancers that over-express human epidermal growth factor receptor (HER2). Triple-negative breast cancer (TNBC) accounts for 10–20% of BC cases. Lacking the overexpression of HER2 and being negative for ER and PR, TNBC does not respond to targeted or hormone replacement therapies. As such, TNBC is an aggressive BC subtype that is associated with poor prognosis (Breastcancer.org), mandating that alternative treatment approaches be sought. In this regard, therapeutic approaches using plant sources have been gaining interest and popularity (Howes, 2018). Contextually, women have an inclination for the use of natural products and herbal remedies as these are claimed to be safer alternatives without significant side effects compared with conventional medicines (Cassidy, 2003). Furthermore, plants have a long history in the treatment of cancer and have been a source of several anticancer drugs (Cragg and Newman, 2005; Buyel, 2018).

*Origanum syriacum L.* is an aromatic perennial shrub native to the Mediterranean region and widely used in culinary practices. It has also been traditionally used in folk medicine to relieve stomach pain and in the treatment of colds and toothaches (Alwafa et al., 2021). In recent years, it has been reported to be rich in bioactive compounds such as flavonoids, glycosides, terpenes, and phenols (Mesmar et al., 2022). These bioactive compounds bestow the plant with various pharmacological properties including antioxidant, anti-inflammatory, anticancer, antimicrobial, and neuroprotective effects, among others (Alwafa et al., 2021). Importantly, its extracts have been documented to inhibit the proliferation of human BC MCF-7 cells (Al-Kalaldeh et al., 2010; Husein et al., 2014) and leukemic TH-1 cells (Ayesh et al., 2014). This prompted the investigation of the effects of the plant in the context of the aggressive TNBC subtype, using MDA-MB-231 BC cells as an *in vitro* model of TNBC.

In this study, we screened the phytochemical constituents of an ethanolic extract of *O. syriacum* (OSEE) and tested its effect on the malignant phenotype of MDA-MB-231 cells, aiming to uncover the possible molecular mechanisms behind its anticancer activity. We report that OSEE has a potent antioxidant activity. Importantly, OSEE inhibited the proliferation of TNBC cells by causing a G_0_/G_1_ phase arrest, concomitant with a decrease of Ki67 levels and an increase of p21 levels. OSEE significantly inhibited MDA-MB-231 cell growth and metastatic properties by inhibiting proliferative signaling, activating suppressors of cell growth, enhancing apoptotic cell-death machinery, reducing migration and invasion of MDA-MB1-cells, in addition to inhibiting angiogenesis in a process that correlated with inhibition of iNOS. Mechanistically, OSEE inhibited STAT3 signaling and activated the p38 MAPK pathway, implicating a crosstalk between p21, p53, iNOS, and reactive oxygen species (ROS).

## 2 Materials and methods

### 2.1 *O. syriacum* ethanolic extract

Leaves of *O. syriacum* were collected from South of Lebanon in the spring season (April-June) of 2020 and 2021. The plant was identified as *Origanum syriacum L*. by Mohammad Al Zein, a plant taxonomist at the Biology Department, American University of Beirut (AUB), and a voucher specimen has been deposited at the Post Herbarium (AUB), under number MSA 2020–1. The leaves were rinsed and air-dried in the dark at room temperature, then ground into a fine powder and suspended in 80% ethanol [20 ml of distilled water and 80 ml of absolute ethanol (Fisher Scientific; U.K)] for 72 h in the dark. The suspension was then filtered, dried using a rotary vacuum evaporator and lyophilized. The obtained powder was dissolved in 80% ethanol at a concentration of 200 mg/ml and stored at 4°C.

### 2.2 Phytochemical analysis

Test for tannins: 5 ml of distilled water was added to 0.5 g of the plant extract and ultrasonicated for 15 min at 80*°*C. The mixture was filtered, cooled down to room temperature, and five drops of 0.1%-FeCl_3_ added to the filtrate. Brownish green or blue-black coloration indicated the presence of tannins (Keo et al., 2017).

Test for resins: 5 ml of distilled water was added to 0.5 g of the plant extract and ultrasonicated for 15 min at 30°C. Then, the mixture was filtered. The presence of resins was indicated by turbidity of the filtrate (Keo et al., 2017).

Test for saponins: 5 ml of distilled water was added to 0.5 g of the plant extract and ultrasonicated for 15 min at 80°C. The mixture was filtered, cooled down to room temperature, and then shaken until the formation of a stable persistent froth, which indicated the presence of saponins (Keo et al., 2017).

Test for phenolic compounds: 0.5 g of the plant extract was mixed with 5 ml of ethanol and ultrasonicated for 15 min at 30°C. The mixture was filtered and 2 ml of distilled water added to the filtrate followed by a few drops of 5%-FeCl_3_. The presence of phenolic compounds was determined by the appearance of a dark green color (Keo et al., 2017).

Test for flavonoids: 1 ml of 2% NaOH solution was mixed with 0.2 g of the plant extract. This produced a concentrated, yellow-colored solution. Then, few drops of diluted acid were added to the mixture, which made the solution colorless, indicating the presence of flavonoids (Mir et al., 2013).

Test for quinones: 0.5 g of the plant extract was added to 5 ml of ethanol and ultrasonicated for 15 min at 30°C. The mixture was filtered and 1 ml of concentrated H_2_SO_4_ was added to 1 ml of filtrate. The appearance of a red color indicated the presence of quinones (Keo et al., 2017).

Test for steroids: 5 ml of ethanol was added to 0.5 g of the plant extract and ultrasonicated for 15 min at 30°C. The mixture was filtered and the filtrate was evaporated to dryness. A few milligrams of the dried extract were dissolved in 1 ml of chloroform and 1 ml of glacial acetic acid, and then 1 ml of concentrated H_2_SO_4_ was added to the side of the test tube and mixed with the solution. The presence of steroids was indicated by appearance of a green color (Keo et al., 2017).

Test for cardiac glycosides: 5 ml of ethanol was added to 0.5 g of the plant extract and ultrasonicated for 15 min at 30°C. The mixture was filtered and the filtrate was evaporated to dryness. A few milligrams of the dried extract were dissolved in 1 ml of glacial acetic acid and few drops of 2%-FeCl_3_, and then 1 ml of concentrated H_2_SO_4_ was added to the side of the test tube. The presence of a brown ring indicated the presence of cardiac glycosides (Keo et al., 2017).

Test for terpenoids: 0.5 g of the plant extract was added to 5 ml of chloroform and ultrasonicated for 15 min at 30°C. The mixture was filtered and 2 ml of concentrated H_2_SO_4_ added to the side of the test tube. The presence of a reddish-brown color indicated the presence of terpenoids (Keo et al., 2017).

Test for anthraquinones: 0.5 g of the plant extract was added to 4 ml of benzene. The mixture was filtered, and 10% ammonia solution was added. After shaking, the presence of a red or violet color indicated the presence of anthraquinones (Basiru et al., 2013).

Test for anthocyanins: 5 ml of ethanol was added to 0.5 g of the plant extract and ultrasonicated for 15 min at 30°C. Then, 1 ml of NaOH was added to 1 ml of the extract and heated for 5 min at 100°C. The presence of a bluish-green color indicated the presence of anthocyanin (Bassal et al., 2021).

Test for essential oils: 5 ml of ethanol was added to 0.5 g of the plant extract and ultrasonicated for 15 min at 30°C. Then, 100 µl of 1 M NaOH was added to the filtrate followed by a few drops of 1 M HCl. The formation of a white precipitate indicated the presence of essential oils (Keo et al., 2017).

### 2.3 LC-MS

#### 2.3.1 Sample preparation

Sample was filtered through 0.22 µm PTFE membrane filters and placed in glass vials for further LC-MS analysis.

#### 2.3.2 LC-MS data acquisition

The LC-MS analysis was performed using a single quadripole LC-MS-2020 mass spectrometer (Shimadzu Corporation, Kyoto, Japan), which was equipped with an electrospray ion source (ESI).

UPLC separation was performed using a Kinetex C18 (1.7 µm, 100 mm × 2.1 mm, Phenomenex, Torrance, CA, United States) column. The column temperature was maintained at 40°C. The injected volume was 10 µL. Water and methanol, both supplemented with 0.1% formic acid, were used as mobile phases.

A stepwise gradient method, presented in Table 1, was used for elution, at a flow rate of 0.4 ml/min.

**TABLE 1:** Chromatographic gradient conditions for the analysis of *O. Syriacum* ethanolic crude extract.

Time (min)	Methanol (%)	Water (%)
0	10.0	90.0
5	20.0	80.0
8	40.0	60.0
11	50.0	50.0
13	60.0	40.0
16	80.0	20.0
17	90.0	10.0
19	10.0	90.0
21	10.0	90.0

MS data were collected in the negative ion mode, over a m/z range of 50–1,500. The parameters of electrospray ionization (ESI) source were set as follows: ESI probe temperature 350°C, DL temperature 250°C, heat block temperature 200°C, ESI probe voltage 4.5 kV, detector voltage 1.6 kV, DL voltage 100 V, Q-array RF voltage 60 V, and nebulizing gas flow 1.5 L/min.

### 2.4 Cell culture

Human breast cancer cells MDA-MB-231 (American Type Culture Collection, Manassas, VA) were maintained in DMEM high-glucose medium supplemented with 10% fetal bovine serum (FBS) (both from Sigma-Aldrich, St. Louis, MO, United States) and 1% penicillin/streptomycin (Lonza, Switzerland) and kept in a humidified chamber (37°C and 5% CO_2_).

### 2.5 Cell viability assay

MDA-MB-231 cells (5 × 10^3^) were seeded in 96-well plates and allowed to grow until they reached 30–40% confluence. The cells were then treated with increasing concentrations of OSEE and incubated for a total period of 72 h. Cell viability was measured by the reduction of 3-(4,5- dimethylthiazol-2-yl)-2,5-diphenyltetrazolium bromide (MTT; Sigma-Aldrich, St. Louis, MO, United States). Cell growth was determined as the proportional viability of the treated cells compared with the ethanol vehicle-treated cells, the viability of which is set to be 100%. In cell viability assays with N-acetyl cysteine (NAC; Sigma-Aldrich, St. Louis, MO, United States), 5 mM NAC was added to the cells for 30 min before OSEE treatment. Assays were performed in triplicate and repeated three times. Data are presented as mean values ± standard error of the mean (SEM).

### 2.6 DPPH (α, α-diphenyl-β-picrylhydrazyl) antioxidant activity assay

The antioxidant activity of the ethanolic extract of *O. syriacum* leaves was measured using the DPPH-radical-scavenging assay as previously described but with some modifications (Moraes-De-Souza et al., 2008). DPPH (cat# D9312, Sigma-Aldrich Co.,) is a free radical used as a colorimetric probe to evaluate the antioxidant properties of plant extracts and constituents: the color of the solution changes from purple to pale yellow. 0.5 ml of different concentrations of OSEE (50, 100, 200, 400, and 600 μg/ml) was mixed with 0.5 ml of DPPH solution (0.5 mM in methanol) and 3 ml of methanol. The blank consisted of 0.5 ml of 80% ethanol, 0.5 ml of DPPH solution and 3 ml of methanol. Mixed samples were then kept in the dark for 30 min and the OD was measured at a wavelength of 517 nm using a spectrophotometer. The DPPH-scavenging activity of each concentration of the extract was calculated using the formula: % radical-scavenging activity = [(OD blank—OD plant extract at each concentration)]/(OD blank)] X 100. Ascorbic acid was used as a standard.

### 2.7 Dihydroethidium staining

MDA-MB-231 cells were seeded in 12-well plates and incubated until they reached 50% confluence. The cells were then treated for 24 h with the indicated concentrations of OSEE; media containing less than 1% ethanol was used as the vehicle control. After incubation, the medium was removed and the cells were washed twice with ice-cold phosphate-buffered saline (PBS). DHE stain (6 μM) was added and the cells were incubated in the dark for 45 min. Then the stain was removed, the cells were washed once with cold PBS, and visualized using a ZEISS Axio Observer.

### 2.8 Microscopic analysis of apoptotic morphological changes

Cells were grown in 6-well tissue-culture plates in the absence or presence of the indicated concentrations of OSEE. Morphological changes characteristic of apoptotic cells were determined after 24 and 48 h using an inverted phase-contrast microscope (objectives 10×, 20×, and 40×).

### 2.9 Flow cytometry analysis of cell cycle

Cells were grown in 10-mm tissue-culture plates for 24 h before the addition of OSEE or ethanol at a concnetration equavlent to that present in OSEE as a vehicle control. After incubation, cells were harvested, washed twice, resuspended in 500 µl PBS, fixed with an equal volume of 100% ethanol, and incubated at −20°C for at least 12 h. Cells were then pelleted, washed twice with PBS and permeabilized in 0.1% Triton X-100/PBS and incubated for 15 min on ice. Afterwards cells were pelleted, resuspended in PBS containing 40 μg/ml propidium iodide and 25 μg/ml RNase A, and incubated at 37°C for 5 min. Cell samples were then analyzed with the BD FACSCanto II Flow Cytometry System (Becton Dickinson) and data acquired using the FACSDiva 6.1 software.

### 2.10 Wound-healing assay

MDA-MB-231 cells were grown in 12-well tissue-culture plates until confluent. A scrape was made through the confluent monolayer using a sterile 200-μL plastic pipette tip. The culture medium was then removed, the cells were washed twice with PBS (Sigma-Aldrich, St. Louis, MO, United States) to remove cellular debris, and incubated at 37°C in fresh medium in the presence or absence of the indicated concentrations of OSEE. Photomicrographs of the wound were taken at baseline (0 h) and for the 4–10 h period considered, using an inverted phase-contrast microscope (objective 10×). The width of the wound was expressed as the average ± SEM between the measurements taken at time zero and the corresponding time points.

### 2.11 Trans-well migration chamber assay

The migratory ability of MDA-MB-231 cells was also assessed with trans-well inserts (8 μm pore size; BD Biosciences, Bedford, MA, United States). Cells were seeded at a density of 1.0 × 10^5^ cells per well, into the upper chamber of the insert, and treated with less than 1% ethanol, as a vehicle control, or the indicated concentrations of OSEE. DMEM supplemented with 10% fetal bovine serum was placed into the bottom wells in the system as a chemo-attractant and then the plates were incubated at 37°C for 24 h. Cells were then washed, and non-penetrating cells were removed from the upper surface of the filter with a sterile cotton swab. Cells that had migrated through to the lower surface of the insert were fixed with 4% formaldehyde, stained with DAPI, and counted under a fluorescence microscope. Assay was repeated three times and data were presented as mean values ± SEM.

### 2.12 Matrigel invasion assay

The invasiveness of the MDA-MB-231 cells was evaluated using a BD Matrigel Invasion Chamber (8-μm pore size; BD Biosciences, Bedford, MA, United States). Briefly, cells were seeded at a density of 1.0 × 10^5^ cells per well, into the upper chamber of the insert, and treated with less than 1% ethanol, as a vehicle control, or the indicated concentrations of OSEE. DMEM supplemented with 10% fetal bovine serum was placed into the bottom wells of the chamber as a chemo-attractant and then incubated at 37°C for 24 h. Cells were then washed, and non-penetrating cells were removed from the upper surface of the filter with a sterile cotton swab. Cells that had penetrated through the Matrigel to the lower surface of the insert were fixed with 4% formaldehyde, stained with DAPI, and counted under a fluorescence microscope. Assay was repeated three times and data were presented as mean values ± SEM.

### 2.13 Adhesion assay

MDA-MB-231 cells were grown in the presence or absence of OSEE for 24 h and then seeded onto collagen-coated 24-well tissue-culture dishes in duplicate. Cells were incubated at 37°C for 1 h and unattached cells were removed by gently washing the wells twice with PBS. The number of adherent cells was determined by the MTT reduction assay, as described above.

### 2.14 Chorioallantoic membrane assay

Fertilized chicken eggs were incubated at 38°C and 60% relative humidity for 10 days. Afterwards, the highly vascularized CAM was dropped by drilling a 1-cm^2^ hole through the eggshell into the air sac. OSEE was then applied onto the CAM to test its effect on blood vessel growth. After 24 h, pictures of the CAM were taken and the angiogenic response was as analyzed using the AngioTool software, which quantifies the length of the vessels and number of junctions.

### 2.15 Whole-cell extracts and western blotting analysis

For whole-cell lysates, cells were washed twice with PBS and lysed in 2% SDS, 60 mM Tris lysis buffer (pH 6.8) and centrifuged at 5,000 *g* for 10 min. The protein concentration of the supernatant was determined using the Bradford protein assay kit (Biorad, Hercules, CA, United States) and 25–30-μg aliquots were resolved by 10% sodium dodecyl sulfate-polyacrylamide gel electrophoresis before being transferred to a polyvinylidene difluoride membrane (Immobilon PVDF; Biorad) and blocked for 1 h at room temperature with 5% non-fat dry milk in TBST (TBS and 0.05% Tween 20). Immunodetection was performed by incubating the membrane with specific primary antibodies at 4°C overnight. Horseradish peroxidase-conjugated anti-IgG was used as secondary antibody and immunoreactive bands were detected using the ECL substrate kit (Thermo Scientific, Rockford, IL, United States), according to the manufacturer’s instructions. All primary and secondary antibodies were purchased from Cell Signaling (Cell Signaling Technology, Inc., Danvers, MA, United States).

### 2.16 Statistical analysis

Results were evaluated using Student’s *t*-test. For the comparison of more than two means, ANOVA was used using one-way ANOVA (with Dunnett’s post hoc test) or two-way ANOVA (with Tukey–Kramer’s post hoc test). Data were presented as mean ± SEMand a *p*-value of <0.05 was considered as statistically significant.

## 3 Results

### 3.1 Phytochemical screening

*O. syriacum* has many primary and secondary bioactive metabolites (Mesmar et al., 2022). Extensive HPLC analyses and the phytochemical bioactives of *O. syriacum* have been reported in several studies (Alma et al., 2003; Dorman et al., 2004; Mesmar et al., 2022). Apigenin, naringenin, rosmarinic acid, carvacrol, carveol thymoquinone, thymol, and caffeic acid are some of the reported molecules (Alma et al., 2003; Dorman et al., 2004; Mesmar et al., 2022). Here we confirmed the presence of several classes of phytochemical compounds in OSEE. Table 2 shows that OSEE contains tannins, phenols, flavonoids, quinones, steroids, terpenoids as well as cardiac glycosides and essential oils.

**TABLE 2:** Phytochemical screening of *O. syriacum* ethanolic crude extract.

Metabolite	*OSEE*
Tannins	+
Resins	+
Saponins	-
Phenols	+
Flavonoids	+
Quinones	+
Sterols and steroids	+
Cardiac glycosides	+
Terpenoids	+
Anthraquinones	-
Anthocyanins	-
Essential oils	+

(−): absent; (+): present.

### 3.2 LC-MS of *O. Syriacum* crude extract

**FIGURE 1:**
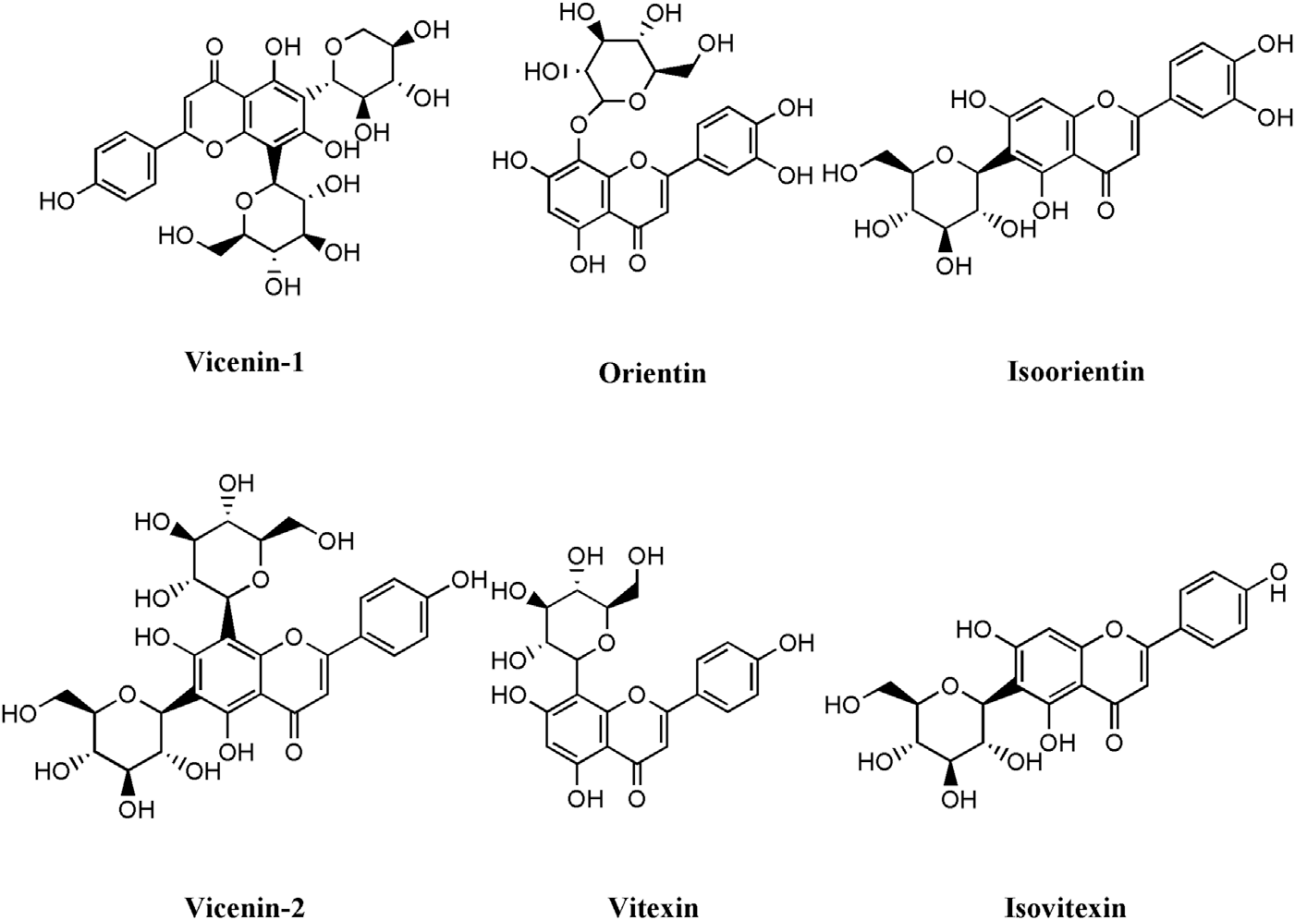
Structures of the compounds identified from *O. Syriacum* ethanolic crude extract using LC-MS.

Metabolites were principally identified by matching masses and retention times of pure standards. Six compounds were identified as shown in Table 3 and Figure 1.

**TABLE 3:** Compounds identified from *O. Syriacum* ethanolic crude extract.

Compound name	Elemental composition	[M-H]^−^ precursor ion (*m/z*)		RT (min)
Vicenin-1	C_26_H_28_O_14_	563.14		5.23
Vicenin-2	C_27_H_30_O_15_	593.15		4.82
Orientin	C_21_H_20_O_11_	447.09		5.60
Isoorientin	C_21_H_20_O_11_	447.09		5.82
Vitexin	C_21_H_20_O_10_	431.09		6.11
Isovitexin	C_21_H_20_O_10_	431.09		6.71

### 3.3 OSEE inhibits the proliferation of MDA-MB-231 breast cancer cells

**FIGURE 2:**
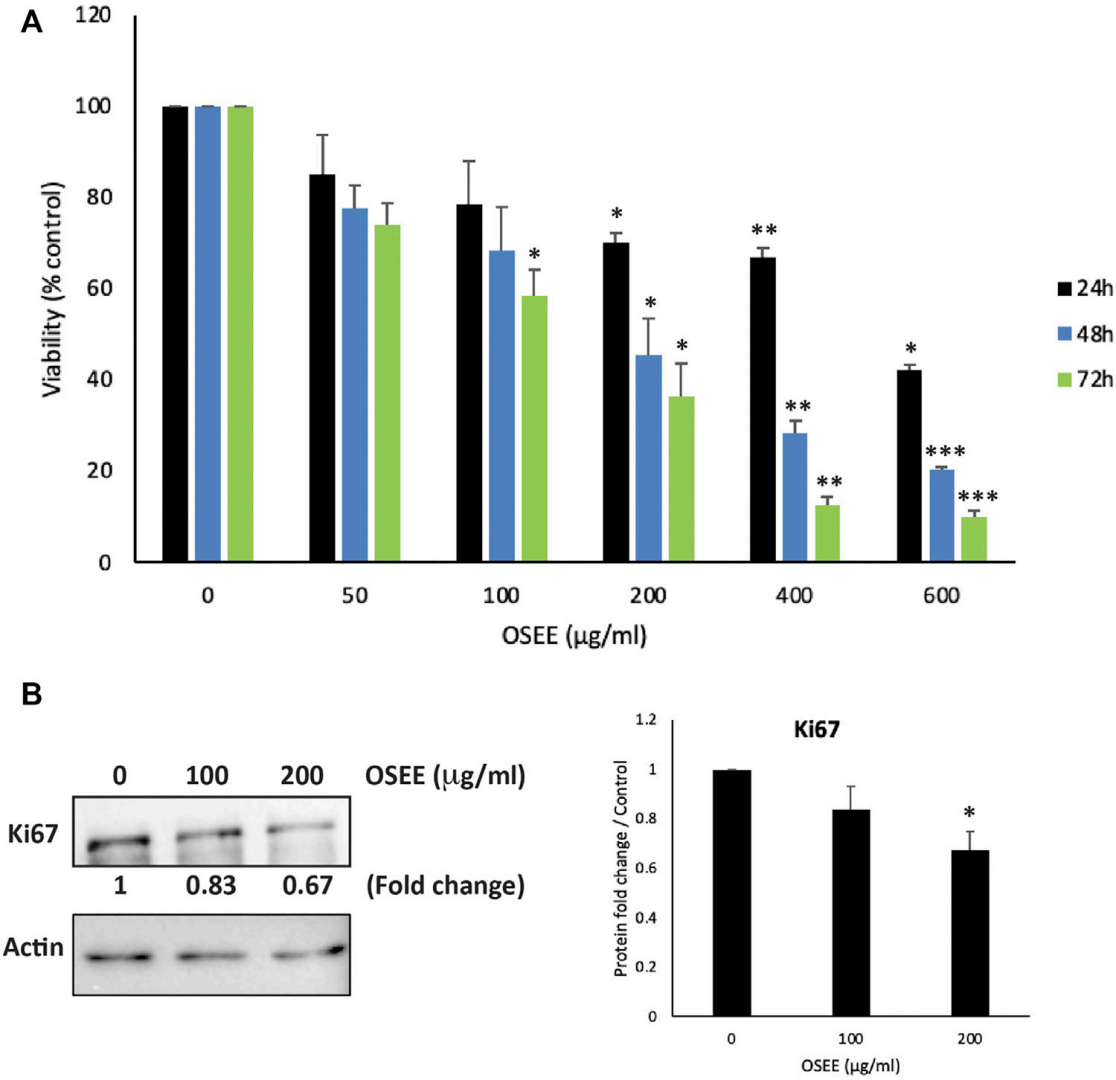
*O. syriacum* inhibits cellular proliferation of MDA-MB-231 breast cancer cells. **(A)** MDA-MB-231 cells were treated or not with the indicated concentrations of *O. syriacum* ethanolic extract (OSEE) for 24, 48, and 72 h. For vehicle control, a concnetration of ethanol equivalent to that present in OSEE was used. Cell viability was monitored using the metabolism-based MTT assay, as described in Materials and Methods. **(B)** MDA-MB-231 cells were incubated for 24 h with and without the indicated concentrations of OSEE. Cells were then lysed, and protein lysates were subject to Western blotting with a Ki67 antibody. Data represent the mean ± SEM of three independent experiments (*n* = 3) carried out in triplicate and expressed as a percentage of the corresponding control cells. Statistical analysis was performed using one-way ANOVA followed by LSD post-hoc test (*p < 0.05, **p < 0.005, ***p < 0.001).

Several of the bioactives reported to be present in *O. syriacum* have been shown to have potent anti-BC effects. Naringenin, apigenin, carvacrol, thymoquinone, thymol, and rosmarinic acid were shown to reduce the malignant phenotype of BC cell lines (Kanno et al., 2005; Demain and Vaishnav, 2011; Lee et al., 2019; Messeha et al., 2020; Sampaio et al., 2021). Knowing that the unfractionated plant extract may often have more potent activities than a single or a few of its phytochemicals, it was thought prudent to investigate the anti-cancerous effects of *O. syriacum* leaves in a triple-negative human BC cell line (TNBC), MDA-MB-231. To this end, we examined the anti-proliferative activity of OSEE against MDA-MB-231 cells. The effect of various concentrations (0, 50, 100, 200, 400 and 600 μg/ml) of the extract on the proliferation of human TNBC MDA-MB-231 cells was assessed at 24, 48, and 72 h of treatment. Results showed that OSEE treatment decreased cell viability in a concentration- and time-dependent manner (Figure 2A). For example, at 48 h of treatment, cell viability using 50, 100, 200, 400 and 600 μg/ml of OSEE to treat MDA-MB-231 cells was 77.4 ± 5.1, 68.4 ± 9.5, 45.5 ± 7.9, 28.2 ± 2.9, and 20.2 ± 6.9% that of control cells, respectively (Figure 2A). The half-maximal inhibitory concentration (IC_50_) was 875, 179.4, and 125.4 μg/ml at 24, 48, and 72 h, respectively. Based on these IC_50_ values, 100 and 200 μg/ml OSEE were used in further experiments.

To confirm the anti-proliferative effects of OSEE, protein lysates from OSEE-treated MDA-MB-231 cells were immunoblotted with an antibody against Ki67, a widely used biomarker for the evaluation of cell proliferation and the prognosis of many cancers. Particularly, Ki67 is highly expressed in TNBC, which is associated with its aggressive pathologic features and poor clinical outcomes (Yang C et al., 2018). Figure 2B, shows that treatment of MDA-MB-231 cells with 100 and 200 μg/ml OSEE caused a remarkable decrease in Ki-67 protein levelsby 0.83- and 0.67-fold, compared with vehicle-treated control cells, respectively (Figure 2B). The decrease in Ki67 protein levels seems to be concentration-dependent (Figure 2B). These data confirm data from Figure 2A, suggesting that OSEE does indeed interfere with the cell proliferation process in MDA-MB-231 cells.

### 3.4 OSEE has potent antioxidant activity and can increase the generation of ROS in MDA-MB-231 cells

**FIGURE 3:**
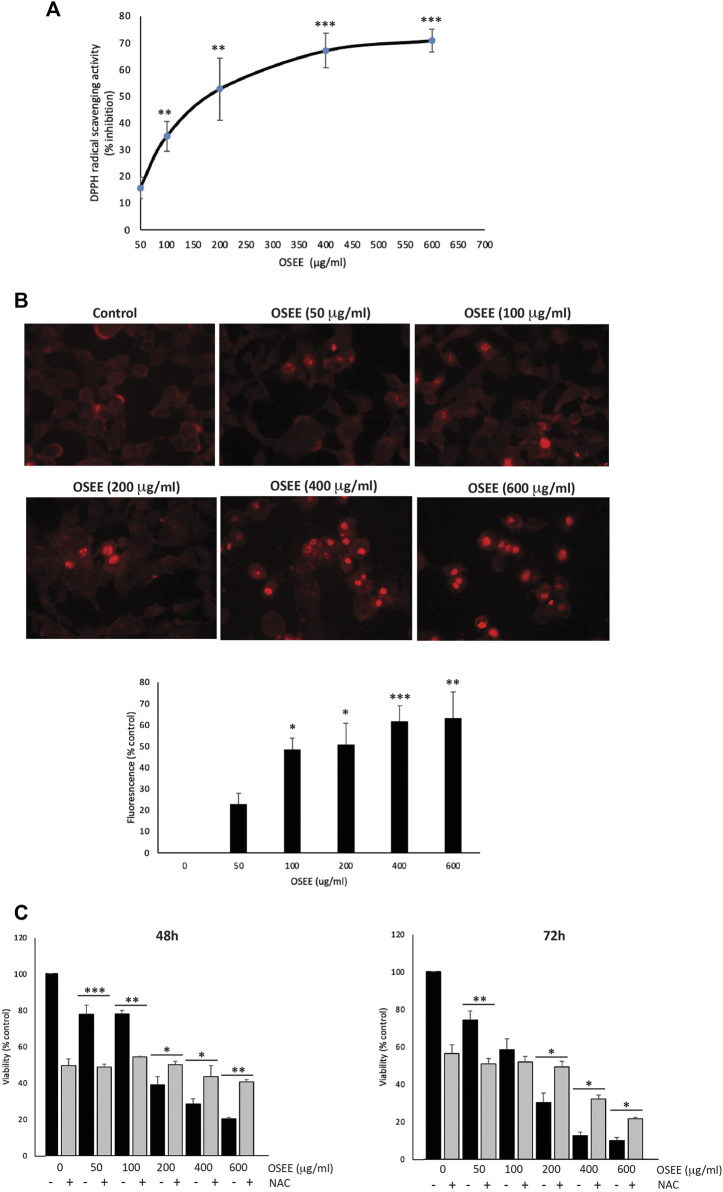
*O. syriacum* has remarkable antioxidant potential and can increase the generation of ROS in MDA-MB-231 cells. **(A)** The antioxidant activity of the indicated concentrations of OSEE was measured by 2,2-di-phenyl-1-picrylhydrazyl (DPPH) radical scavenging capacity assay as described in Materials and Methods. Data represent the means ± SEM of three independent experiments. **(B)** Fluorescent images of dihydroethidium (DHE)-stained MDA-MB-231 cells. Cells were treated with and without the indicated concentrations of OSEE for 24 h and stained with DHE, as indicated in Materials and Methods, to measure intracellular ROS production. **(C)** MDA-MB-231 cells were pre-treated with NAC (10 mM) for 30 min and then with OSEE at the indicated concentrations. Cell viability was measured using the MTT assay at the indicated time points. OSEE-treated cells without NAC pre-treatment were used for comparison. Values represent the means ± SEM of three independent experiments performed in triplicates and expressed as percentage of vehicle-treated control cells (*p < 0.05, **p < 0.005, ***p < 0.001).

ROS are implicated in many aspects of health and disease, including signaling processes. There is a delicate balance between oxidative stress and antioxidant mechanisms inside the cell, ensuring that physiological functions are maintained, and proper defense mechanisms are in place. Any disturbance of this balance may lead to pathological outcomes. Notably, both ROS and antioxidants have been shown to play either anti- or pro-cancerous roles. Indeed, phytochemicals and other natural products have been reported to act as anti- or pro-oxidant agents, depending on the context, in a biphasic and concentration-dependent manner. For example, dietary supplementation with the antioxidant N-acetyl cysteine (NAC) can promote cancer progression and metastasis (Liou and Storz, 2010; Chio and Tuveson, 2017; Shaito et al., 2020). *O. syriacum* has been reported to contain many bioactive molecules with high antioxidant potential, such as polyphenols. In this study, OSEE antioxidant potential was evaluated *in vitro* using the DPPH-radical-scavenging assay. OSEE exhibited significant free-radical-scavenging activity which was concentration-dependent (Figure 3A). Despite this significant antioxidant-radical-scavenging activity of OSEE in the test tube *in vitro* (Figure 3A), we tested the effect of OSEE on ROS generation in MDA-MB-231 cells in culture. Indeed, MDA-MB-231 cells treated with increasing concentrations of OSEE, showed increased DHE fluorescence as the concentration of OSEE increased (Figure 3B), indicating that OSEE increases the levels of ROS generation in MDA-MB-231 cells.

Reactive oxygen species function like a double-edged sword in cancer progression, depending on their concentration in the cell and the stage of cancer. ROS have been reported to either enhance tumorigenesis and promote tumor progression by causing DNA damage and inducing pro-oncogenic pathways, or to induce cell death and apoptosis of cancer cells (Shaito et al., 2020; Oyenihi et al., 2022; Oyenihi et al., 2022). To investigate whether OSEE exerts anti-proliferative effects on MDA-MB-231 cells through ROS generation, MDA-MB-231 cells were first pre-treated with NAC to dampen ROS generation, followed by treatment with OSEE at various concentrations. Cell proliferation and viability were then assessed for a period of 3 days. NAC, acting as a ROS scavenger, can by itself inhibit proliferation of MDA-MB-231 cells at 24 h (data not shown), 48 h, and 72 h of treatment (Figure 3C). Figure 3C also shows that treatment with NAC augmented the inhibition of proliferation of MDA-MB-231 cells treated with lower concentrations of OSEE (50 and 100 μg/ml). However, NAC treatment was not able to blunt the inhibition of proliferation of MDA-MB-231 cells induced by higher concentrations of OSEE (200, 400 and 600 μg/ml); on the contrary, OSEE attenuated NAC-induced inhibition of proliferation (Figure 3C). These data indicate that the anti-proliferative effects of OSEE may depend on the levels of ROS generation inside the cell, confirming the biphasic concentration-dependent effects reported for other natural antioxidants.

### 3.5 OSEE induces cell-cycle arrest of MDA-MB-231 cells at G_0_/G_1_ phase

**FIGURE 4:**
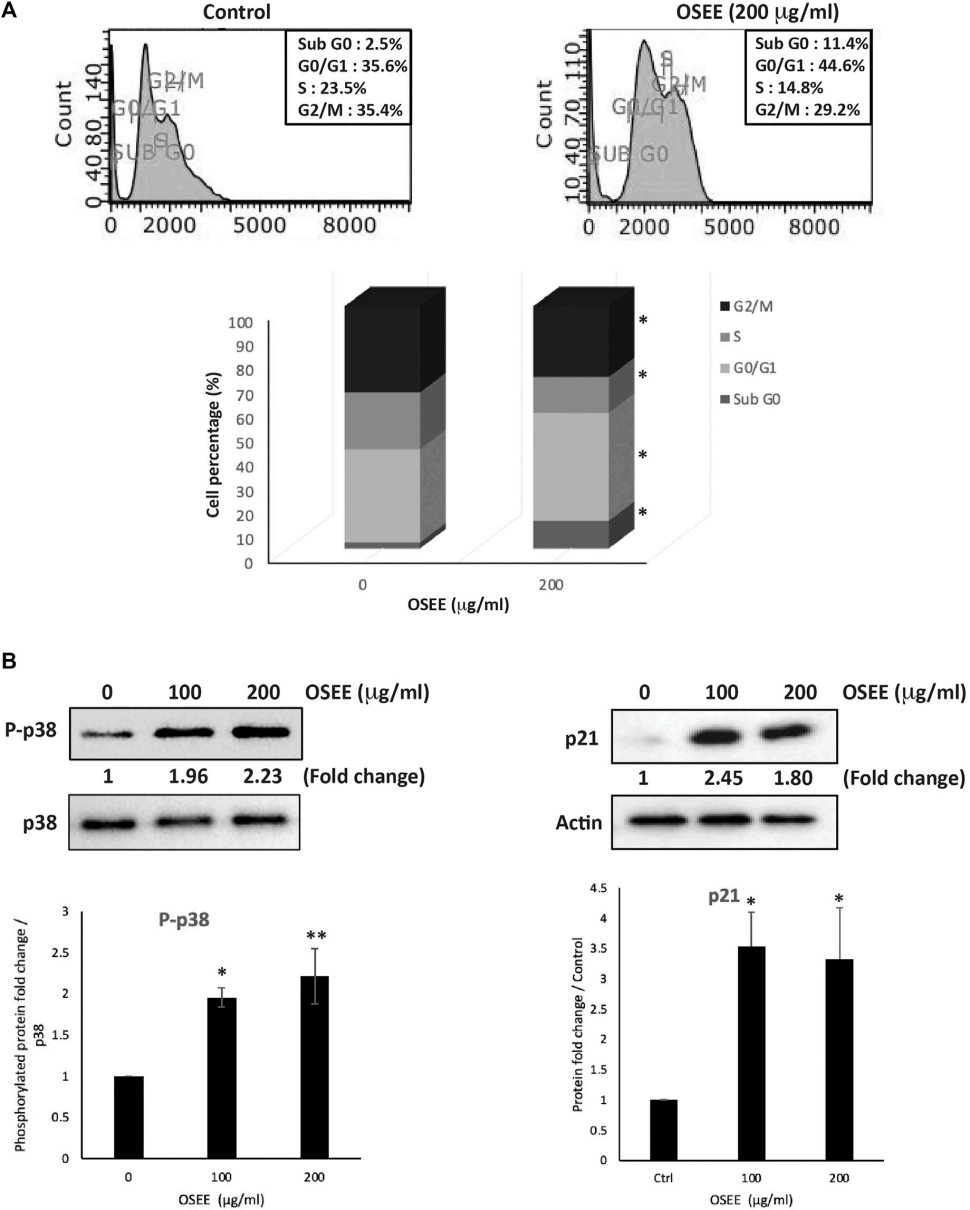
*O. syriacum* induces G_0_/G_1_ cell cycle arrest in MDA-MB-231 cells. **(A)** MDA-MB-231 cells were incubated with OSEE (200 μg/ml) or ethanol as a vehicle control for 24 h. Cells were then harvested, fixed, stained with propidium iodide, and analyzed by flow cytometry as described in Materials and Methods. Data represent the mean ± SEM of three independent experiments. Statistical analysis was performed using one-way ANOVA (*p < 0.05, **p < 0.005) **(B)** MDA-MB-231 cells were treated with or without increasing concentrations of OSEE for 24 h. Proteins were then extracted and the levels of phosphor-p38 and p21 were analyzed by Western blotting, with total-p38 and β-actin as loading controls, respectively. Data represent the mean ± SEM of three independent experiments (* denotes a *p* < 0.05 and ** denotes a *p* < 0.005).

To investigate the mode of the anti-proliferative effect induced by OSEE in MDA-MB-231 cells, the cell-cycle distribution of these cells was assessed, using PI staining followed by flow cytometry, at 24 h of treatment with 200 μg /ml OSEE. Figure 4A, shows that OSEE induced an arrest at the G_0_/G_1_ phase of the cell cycle. The percentage of cells in G_0_/G_1_ phase increased in OSEE-treated cells (44.6 ± 0.8 vs 36.5 ± 2.1 in control cells), accompanied by a concomitant decrease in the percentage of cells in the S phase (14.8 ± 1.0 vs 23.5 ± 1.3 in control cells), suggesting that OSEE triggers a G_1_ phase arrest and inhibits entry into the S phase (Figure 4A). The cell cycle data also revealed that untreated control cells hardly exhibit any sub-G_0_ DNA (Figure 4A). Treatment of cells with OSEE caused a significant increase in the sub-G_0_ cell population, indicative of apoptosis (Figure 4A).

The p38 MAPK (mitogen-activated protein kinase) pathway has been widely associated with anti-proliferative functions by regulating cell cycle progression and inducing apoptosis to maintain cellular homeostasis (Lafarga et al., 2009; Martínez-Limón et al., 2020). Western blotting analysis of the levels of the active phosphorylated form of p38 indicated significant activation of p38 following treatment of MDA-MB-231 cells with 100 or 200 μg/ml OSEE (1.96 ± 0.11- and 2.23 ± 0.33-fold increases, respectively) (Figure 4B). Our results also show that the levels of a downstream effector of p38, the cell cycle regulator protein p21 (Lafarga et al., 2009), which inhibits progression of the cell cycle, increased remarkably upon treating the cells with OSEE (Figure 4B), further confirming the cell cycle data.

### 3.6 OSEE induces intrinsic apoptosis in MDA-MB-231 cells

**FIGURE 5:**
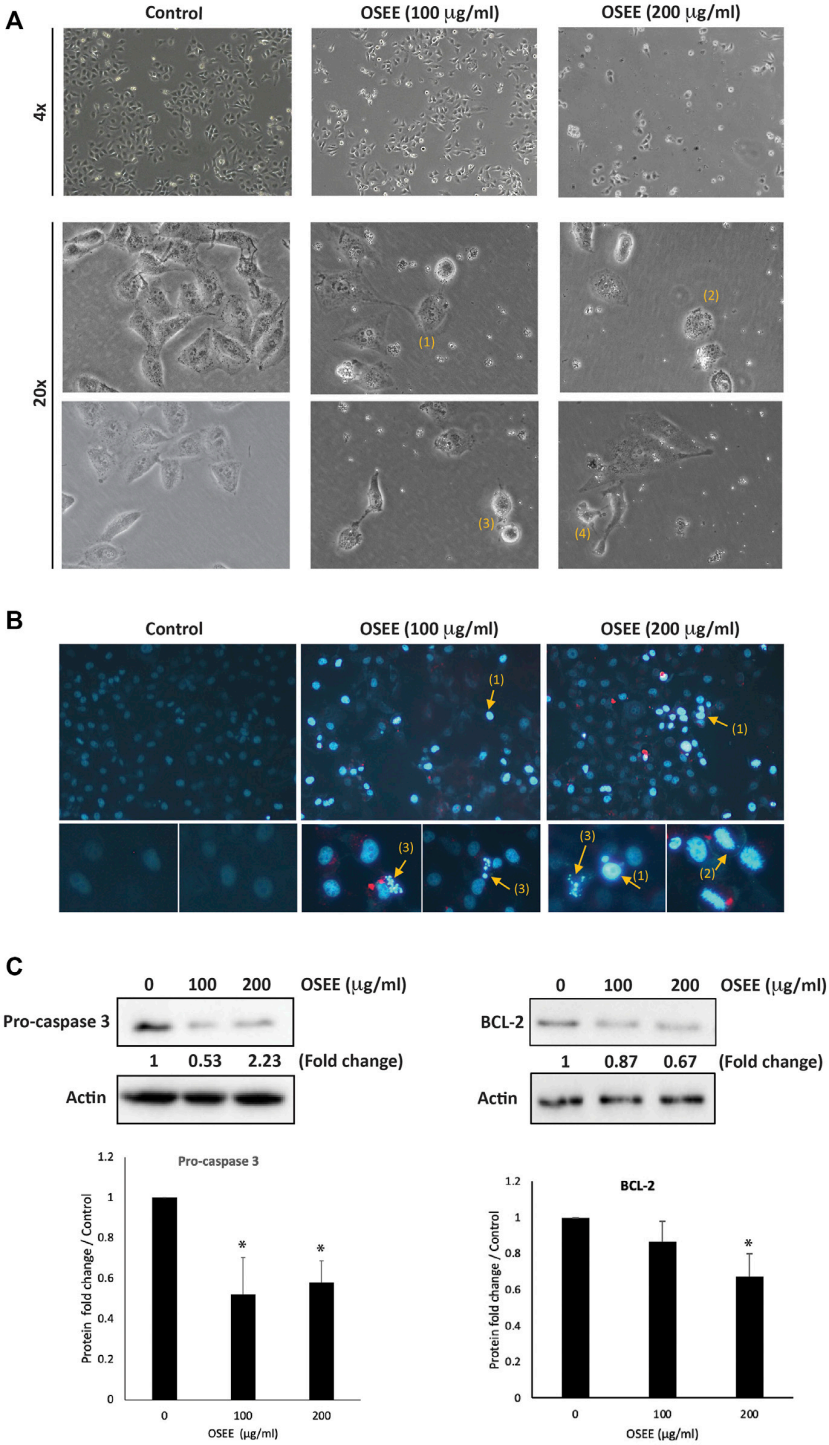
*O. syriacum* induces apoptosis in MDA-MB-231 cells. **(A)** MDA-MB-231 cells were treated with and without the indicated concentrations of OSEE for 24 h. Morphological changes were observed by light microscopy. Arrows show (1) cell shrinkage, (2) membrane blebbing, (3) apoptotic bodies, and (4) echinoid spikes. **(B)** Cells were incubated with OSEE at the indicated concentrations for 24 h and stained with 4′,6-diamidino-2-phenylindole (DAPI) to visualize nuclei. Nuclear morphological changes and apoptosis were then assessed using a fluorescence microscope. Arrows indicate (1) condensation of nuclear material, (2) cell swelling and chromatin lysis, and (3) apoptotic bodies. **(C)** Cells were treated with and without the indicated concentrations of OSEE for 24 h. Protein levels of pro-caspase 3 and BCL-2 were determined by Western blotting. Immunoblotting for β-actin was used as a loading control. Data represent the mean of three ± SEM independent experiments (*n* = 3). (* denotes a *p* < 0.05).

To follow up on sub-G_0_ cell cycle data and to confirm the apoptosis status in OSEE-treated MDA-MB-231 cells, the cells were examined 24 h after treatment with OSEE. Analysis of OSEE-treated cells using an inverted phase-contrast microscope showed an OSEE concentration-dependent decrease in the total number of cells per microscopic field, and the appearance of apoptotic cells characterized by cell shrinkage, membrane blebbing, and nuclear abnormalities (Figure 5A). Further analysis of OSEE-treated and DAPI-stained cells showed condensation of nuclear material, chromatin lysis, and the presence of apoptotic bodies, all indicative of possible induction of apoptosis by OSEE treatment (Figure 5B).

Central to the execution of apoptosis is the processing of procaspase-3 to the active form, caspase-3. To gain further insight into the mechanism of apoptosis induced by OSEE, we examined the levels of procaspase-3 protein. The results showed a significant decrease in procaspase-3 levels in cells treated with 100 and 200 μg/ml OSEE (0.58 ± 0.18- and 0.52 ± 0.11-fold reductions, respectively), suggesting that OSEE enhanced the proteolytic processing of procaspase-3, and consequently augmented caspase activation and induced the intrinsic apoptotic cascade (Figure 5C).

The B-cell lymphoma 2 antiapoptotic protein, BCL-2, also plays an important role in the intrinsic apoptosis pathway and has been shown to contribute to chemoresistance in many cancers (Yip and Reed, 2008), implying that targeting BCL-2 could have a potential role in the treatment of TNBC. In our study, OSEE-treated cells showed a decrease in BCL-2 protein levels in a concentration-dependent manner, achieving a significant difference from the control at 200 μg/ml of OSEE (Figure 5C). These data further confirm that OSEE induces cell death by targeting apoptotic mechanisms.

### 3.7 OSEE inhibits the STAT3 signaling pathway

**FIGURE 6:**
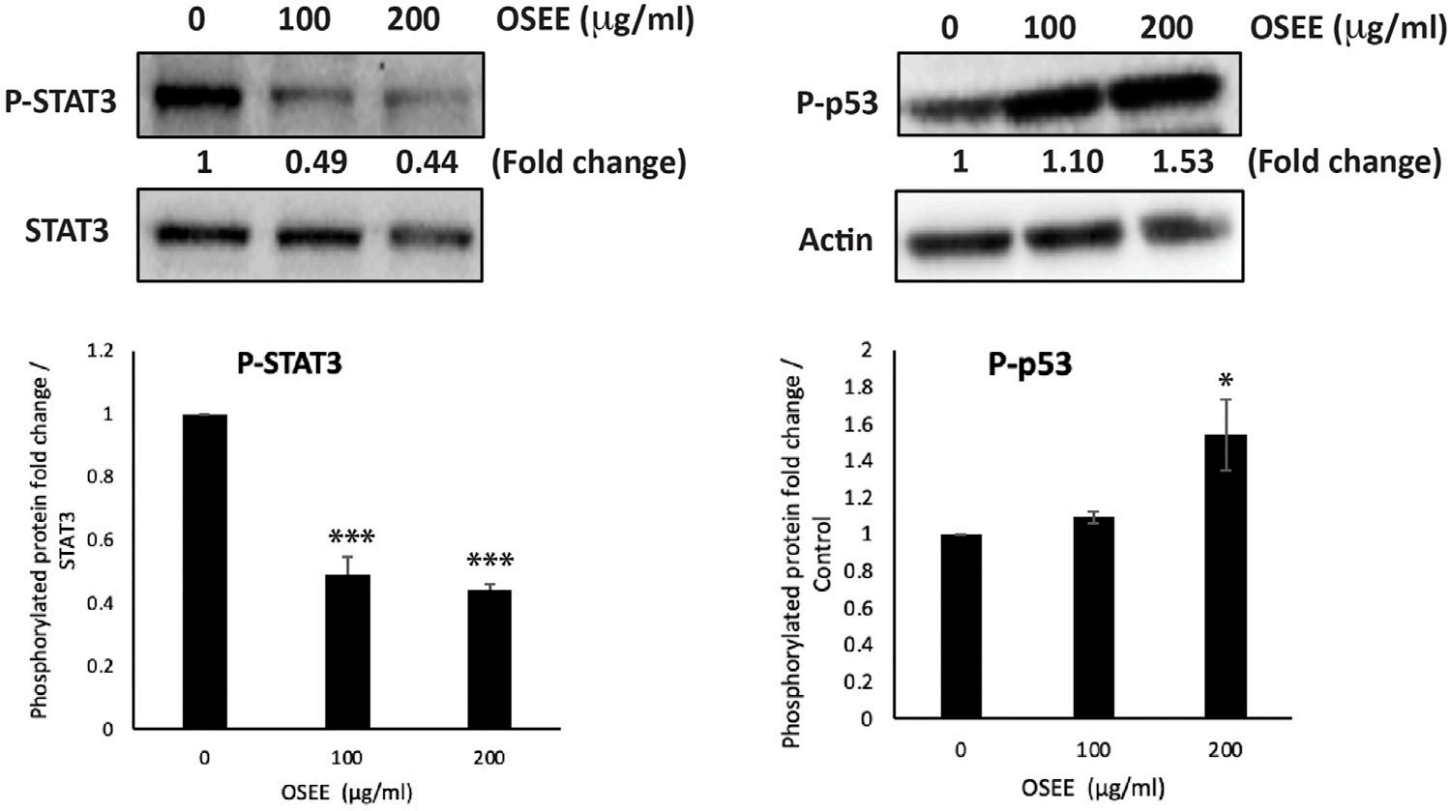
*O. syriacum* inhibits the STAT3 signaling pathway in MDA-MB-231 cells. MDA-MB-231 cells were treated with and without the indicated concentrations of OSEE for 24 h and protein lysates were examined for the phosphorylation of STAT3 and levels of phospho-p53 by Western blotting. Values represent the mean ± SEM of three independent experiments (*n* = 3). *p < 0.05 and ***p < 0.001.

To analyze the molecular signaling behind OSEE-induced apoptosis, we assessed the expression levels of p53 and STAT3 (signal transducer and activator of transcription 3 protein), in MDA-MB-231 cells treated with OSEE at 100 μg/ml and 200 μg/ml for 24 h. STAT3 is a transcription factor with established oncogenic properties. It is activated by phosphorylation, inducing its dimerization and subsequent translocation to the nucleus, where it reportedly has been shown to inhibit endogenous expression of p53 protein. Our results showed a significant decrease in the phosphorylation of STAT3 by 0.49 ± 0.05- and 0.44 ± 0.02-fold in cells treated with 100 μg/ml and 200 μg/ml OSEE, compared to vehicle control-treated cells (Figure 6). Moreover, a significant increase was observed in the phosphorylation of p53 upon treatment of MDA-MB-231 cells with 200 μg/ml of OSEE (Figure 6). These data suggest that OSEE inhibits STAT3 signaling, resulting in the activation of p53, and therefore induction of intrinsic apoptosis mediated by caspase-3.

### 3.8 OSEE increases the aggregation of MDA-MB-231 cells

**FIGURE 7:**
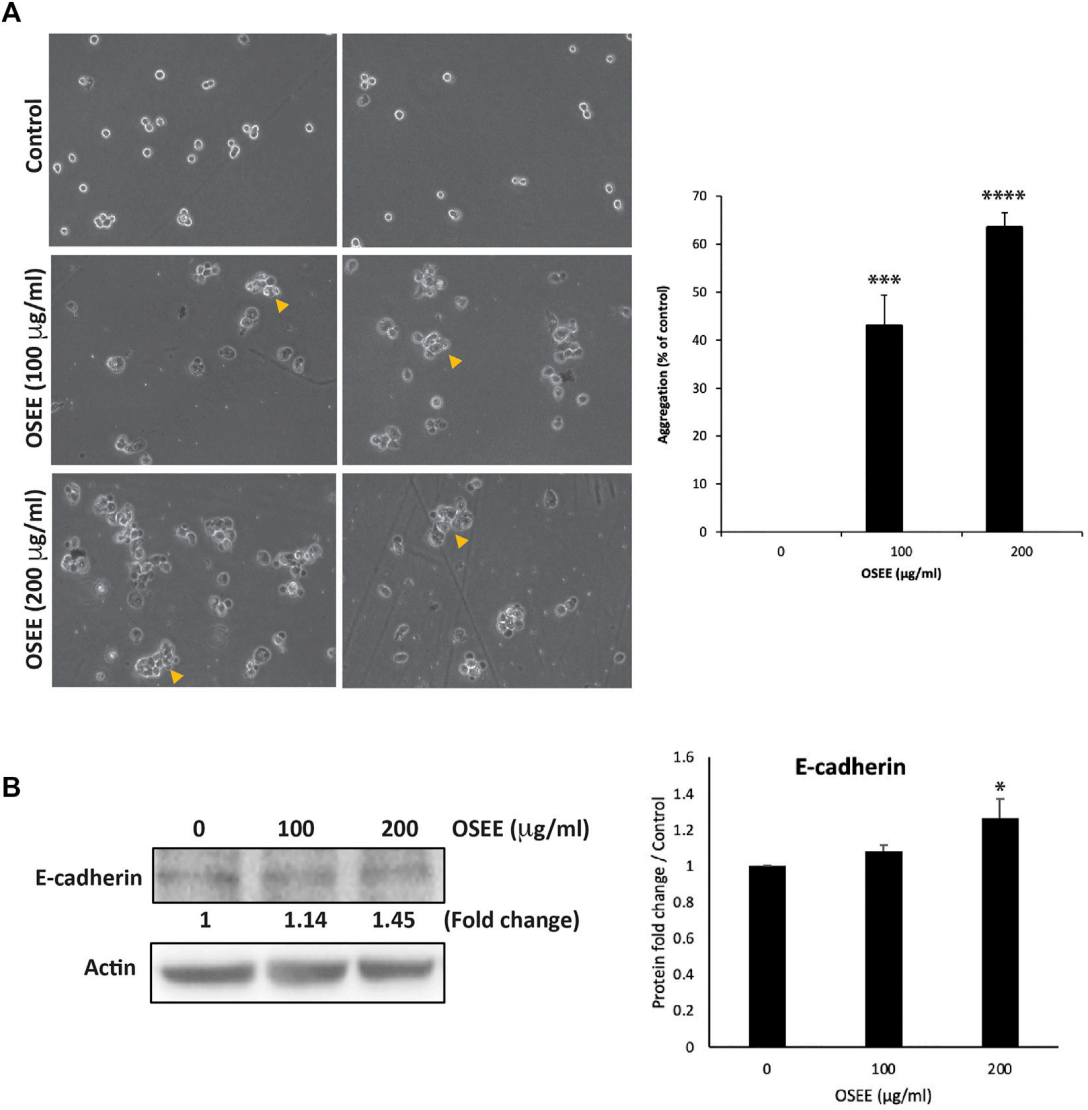
*O. syriacum* increases the cell-cell aggregation of MDA-MB-231 cells. **(A)** MDA-MB-231 cells were incubated with and without the indicated concentrations of OSEE and subjected to a cell-aggregation assay as described in Materials and Methods. Micrographs of cells were taken after 1 h and the percentage of cell-cell aggregates was measured using the following equation: % aggregation = (1 – Nt/Nc) x 100, where Nt is the number of single cells in the control and Nc is the number of single cells in the treated sample. **(B)** MDA-MB-231 cells were incubated with and without the indicated concentrations of OSEE for 24 h, and whole-cell protein lysates were analyzed for E-cadherin protein levels by Western blotting. β-actin was used as a loading control. Data represent the mean ± SEM of three independent experiments (*n* = 3). *p < 0.05, ***p < 0.001, ****p < 0.0001.

Epithelial-mesenchymal transition (EMT) is a complex cellular program and a hallmark of the progression of tumor cells towards metastasis. During EMT, epithelial cells acquire a mesenchymal phenotype, characterized by the loss of cell-cell adhesion and an increase in their migratory and invasive properties. MDA-MB-231 cells have undergone a high degree of EMT, and a drug that is designed for the treatment of TNBC is expected to reverse EMT by allowing the cells to regain their epithelial properties such as cell-cell adhesion. To this end, we evaluated the effect of the OSEE extract on the cell-cell adhesion properties of MDA-MB-231 cells in suspension in a cell-aggregation assay. Figure 7A shows that OSEE caused a concentration-dependent increase in cell-cell aggregates compared to the control cells, with significant 43 ± 6.4 and 63.5 ± 3% increases, 1 h after OSEE treatment at 100 μg/ml and 200 μg/ml, respectively.

Cadherins are adhesion receptors that mediate homotypic cell-cell adhesion, and the loss of E-cadherin-mediated cell-cell contact is associated with malignant transformation by inducing EMT, leading to tumor metastasis. Here, MDA-MB-231 cells treated with 100 μg/ml and 200 μg/ml of OSEE showed an increase in E-cadherin protein levels in a concentration-dependent manner, by 1.14 ± 0.03- and 1.45 ± 0.11-fold that of untreated control cells, respectively (Figure 7B).

### 3.9 OSEE reduces the migration and the invasive properties of MDA-MB-231 cells

**FIGURE 8:**
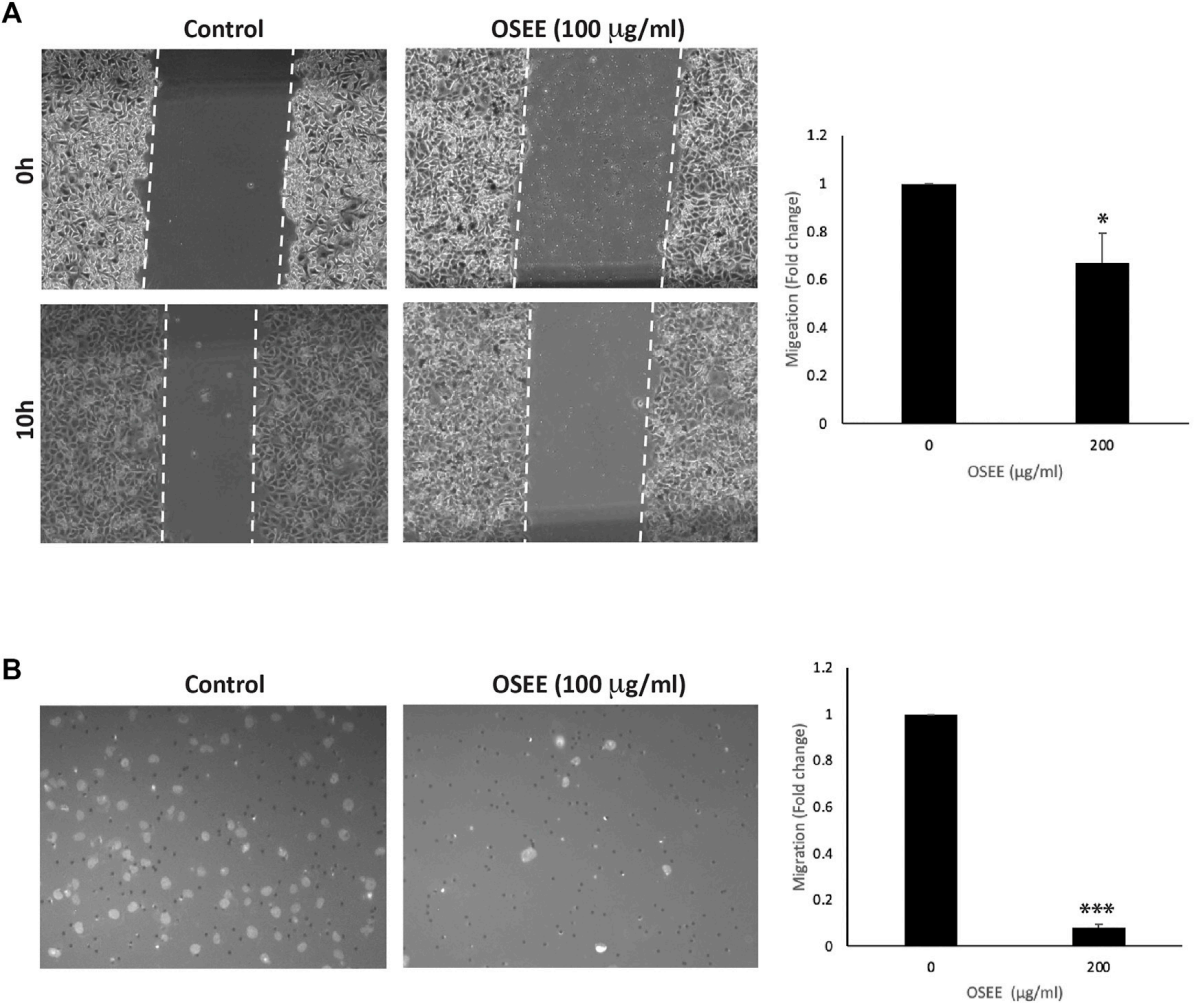
*O. syriacum* inhibits the migration of MDA-MB-231 cells. **(A)** A confluent culture of MDA-MB-231 cells was wounded by scratching with a pipette tip. The cells were then incubated with and without the indicated concentrations of OSEE. After 10 h, the wound was photographed using an inverted phase-contrast microscope and then measured and analyzed. Values represent the fold change in migration compared to vehicle control cells. **(B)** MDA-MB-231 cells were incubated overnight with and without the indicated concentrations of OSEE in Boyden chamber trans-well inserts as described in Materials and Methods. Migrating cells at the bottom of the chamber were stained with DAPI, imaged, and then counted and analyzed. Values represent the average of three independent experiments and are represented as mean ± SEM (*p < 0.05, ***p < 0.001).

Having established that OSEE affects cell-cell interactions, we assessed the effect of the extract on cell migration, a main characteristic of the malignant phenotype. Although cell migration is essential in many physiological processes such as wound repair, tissue formation, and proper immune response, its deregulation contributes to the initial steps of cancer metastasis as cells spread away from the primary tumor site. The effect of OSEE on the migration of MDA-MB-231 cells was examined using assays for wound healing and trans-well migration. Figure 8A shows that OSEE decreased the migration of MDA-MB-231 cells, as demonstrated by a decrease in the ability of those cells to migrate and fill the scratched area. For example, 10 h after the scratch was applied to a confluent monolayer, the migration of MDA-MB-231 cells treated with OSEE at 200 μg/ml was 0.67 ± 0.4-fold that of the control cells (Figure 8A). This inhibition of migration was further confirmed using the trans-well migration assay: OSEE caused a marked decrease in the cell migration ability of MDA-MB-231 cells since only 7.8 ± 0.2% of cells were able to cross from the upper to the lower chamber (Figure 8B).

**FIGURE 9:**
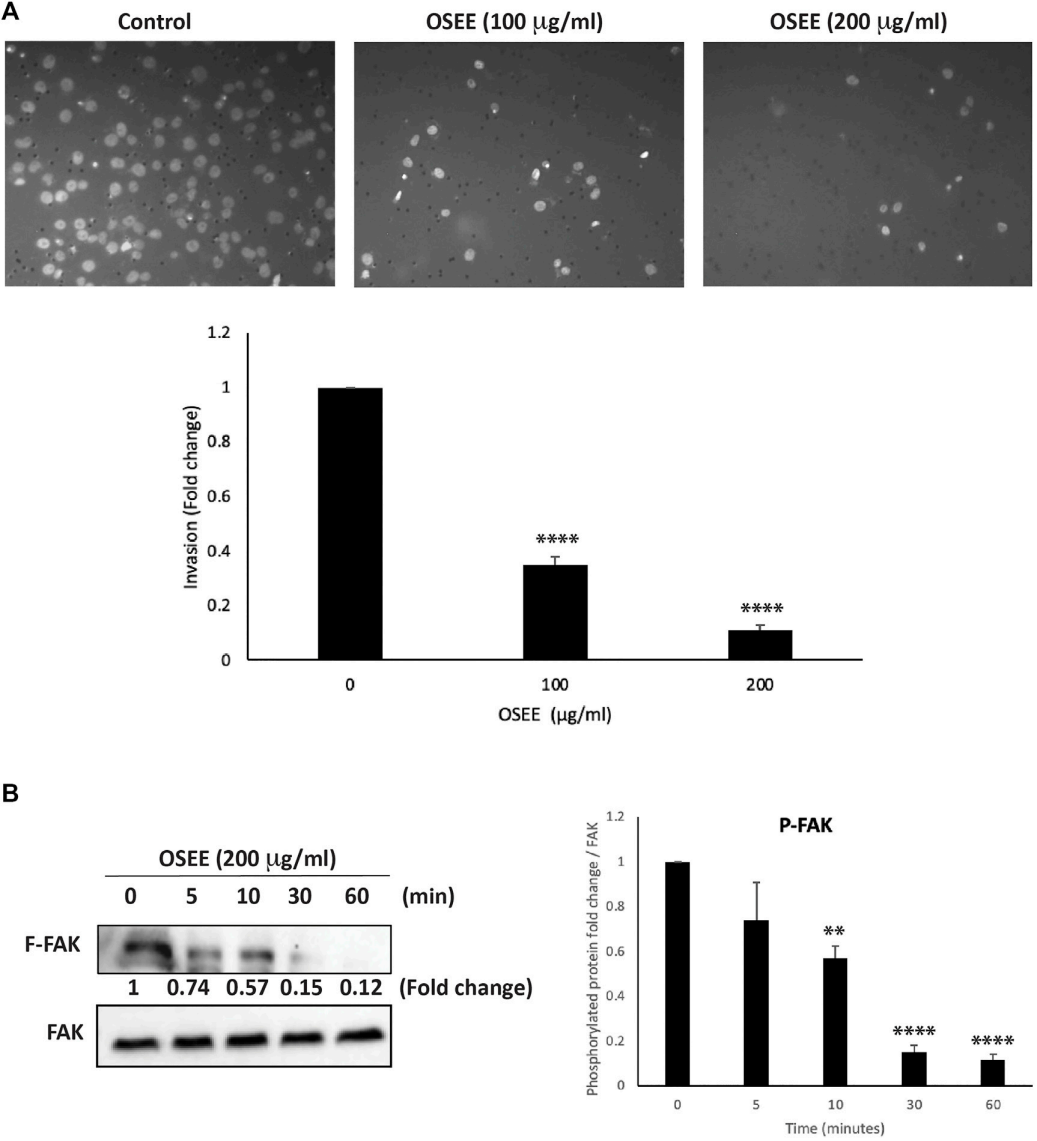
*O. syriacum* reduces the invasive potential of MDA-MB-231 cells. **(A)** MDA-MB-231 cells were incubated overnight with and without the indicated concentrations of OSEE inBoyden chamber trans-well inserts pre-coated with Matrigel as described in Materials and Methods. Cells that invaded the Matrigel were stained with DAPI, imaged, and then counted and analyzed. Values represent the fold change in migration of the ethanol-treated control. The experiment was repeated three times (*n* = 3) and data represent the mean ± SEM (**p < 0.005, ****p < 0.0001). **(B)** Cells were treated with and without 200 μg/ml OSEE at different time points and the phosphorylation of FAK was assessed at hose points by Western blotting, using total-FAK as loading control. The Western blot is representative of three independent experiments (*n* = 3). **p < 0.005, ****p < 0.0001.

Cell invasion is an integral component of the early stages of cancer metastasis, highlighting the ability of cancer cells that have spread away from the primary tumor site to invade secondary sites of metastasis. To this end, we examined the effect of OSEE on the invasive potential of MDA-MB-231 cells using matrigel-coated trans-well chambers in the presence or absence of OSEE (100 μg/ml and 200 μg/ml). The results showed that the number of cells that invaded the matrigel matrix to reach the bottom chamber was significantly reduced by OSEE treatment, by as much as 65 ± 3.1% and 89 ± 1.7%, for 100 μg/ml and 200 μg/ml, respectively, compared with the control (Figure 9A). This reduction seems to be concentration-dependent and suggests that OSEE effectively reduces the invasive potential of MDA-MB-231 cells.

Focal adhesion kinase (FAK) has a major role in facilitating and promoting the migration and invasiveness of tumor cells (Luo and Guan, 2010; Tai et al., 2015; Lai et al., 2018; Shen et al., 2018). Here we report that 200 μg /ml OSEE caused a 0.74 ± 0.17-fold decrease in the phosphorylation of FAK within 5 min of treatment (Figure 9B). The decrease was significant as early as 10 min and was 0.12 ± 0.02-fold that of control levels after 1 h of treating MDA-MB-231 cells with OSEE. By impacting the migratory and invasive properties of MDA-MB-231 cells, OSEE can potentially reduce metastasis, the main cause of poor prognosis of TNBC tumors.

### 3.10 OSEE reduces the levels of iNOS and inhibits angiogenesis *in ovo*

**FIGURE 10:**
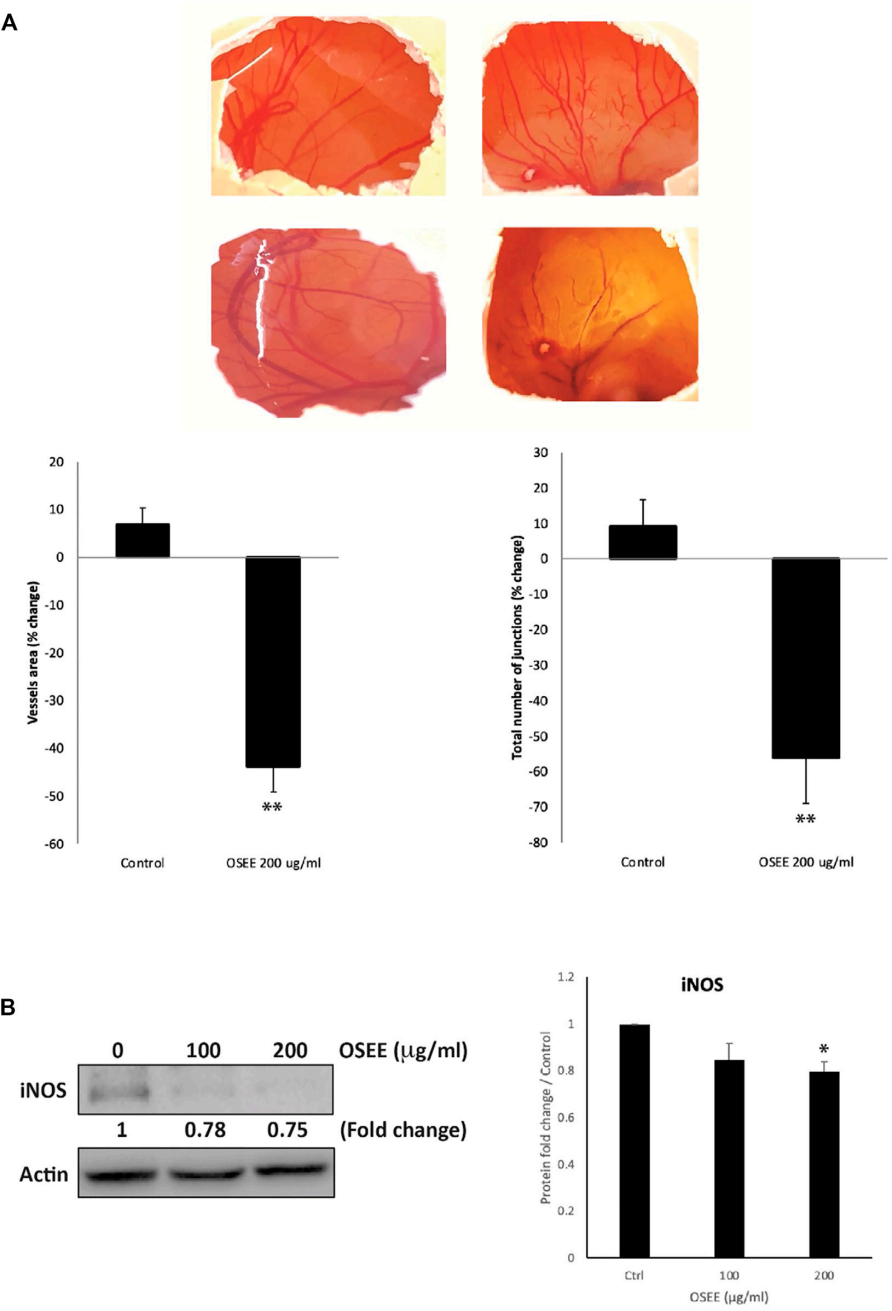
*O. syriacum* inhibits angiogenesis in ovo and reduces iNOS levels in MDA-MB-231 cells. **(A)** OSEE was applied to the chorioallantoic membrane (CAM) of fertilized chicken eggs as described in Materials and Methods. Upper panel of **(A)** shows images of CAM acquired 24 h later to score the angiogenic response. Lower panel of **(A)** shows analysis of the acquired images. Total vessel length and total number of junctions were quantified in both the OSEE-treated and control CAMs using the AngioTool software and represented as percentage change with respect to the control (* denotes *p* < 0.05 and **p < 0.01). **(B)** The protein levels of iNOS were determined by Western blotting in MDA-MB-231 cells treated with or without the indicated concentrations of OSEE for 24 h. The Western blot is representative of three independent experiments. The bar graph represents the quantification of three independent Western blots and the data represent the mean ± SEM of three independent experiments (*n* = 3). *p < 0.05.

Angiogenesis plays a crucial role in tumor growth and metastasis by providing oxygen and nutrients to proliferating cells through the formation of new blood vessels. To test the effect of OSEE on angiogenesis, the chick-embryo chorioallantoic-membrane (CAM) assay was performed. The findings demonstrated that 200 μg/ml of OSEE applied to the surface of the highly vascularized CAM membrane for 24 h caused a significant inhibition of new blood vessel formation (a reduction of 44 ± 5.3%, compared to the control) and a decrease in the number of junctions (a decrease of 56 ± 12.8% compared to the control) (Figure 10A).

Nitric oxide is a main mediator of angiogenesis. Hence, the anti-angiogenic potential of OSEE was further investigated by testing its effect on cytokine-induced expression of inducible nitric oxide synthase (iNOS), a main producer of nitric oxide. Our results showed that OSEE treatment at 100 μg/ml and 200 μg/ml caused a significant decrease in iNOS levels by 0.78 ± 0.06- and 0.75 ± 0.02-fold, respectively, compared to the control (Figure 10B). This indicates that OSEE interferes with the production of nitric oxide, leading to a reduction of angiogenesis.

## 4 Discussion

Breast cancer (BC) cells sustain proliferative signaling, evade growth suppressors, and resist cell death, providing them with a growth advantage over normal cells. In addition, cancer cells are highly migratory, can activate invasion, and induce angiogenesis. Therefore, new cancer therapeutic strategies have focused on compounds with multiple targets or on combination approaches that mix or design hybrid compounds, particularly to treat aggressive hard-to-treat cancers such as TNBC (Kucuksayan and Ozben, 2017). Plants are rich in secondary metabolites with strong antitumor functions, including terpenoids, phenolics, and alkaloids. Our study confirmed that *O. syriacum* contains several classes of these phytochemical compounds, e.g., phenols, flavonoids, quinones, steroids, terpenoids, tannins, cardiac glycosides and essential oils. This is in agreement with other studies showing that *O. syriacum* ethanolic extract contains terpenoids (Kamel et al., 2001), flavonoids, carotenoids, and phenols such as thymol and carvacrol (El-Moneim et al., 2014; Alonazi et al., 2021). These plant-derived natural compounds have been gaining more attention as therapeutic options because of their ability to evade resistance by the cancer cells while producing minimal side effects. In fact, natural compounds can solely target cancer cells, or can complement the effects of other chemotherapeutic drugs by sensitizing cancer cells and modulating drug-drug interactions (Scaria et al., 2020). Many natural compounds have been developed into staple anticancer drugs such as the antimitotic plactitaxel from *Taxus brevifolia* and vinblastine from *Catharanthus roseus*, pro-apoptotic pomiferin from *Maclura pomifera* and *Dereeis Malaccensis*, and anti-angiogenic flavopiridiol from *Dysoxylum binectariferum Hook. f* and combretastatin A-4 phosphate from *Combretum caffrum*, among others (Greenwell and Rahman, 2015; Hassan, 2019). Plants from the *Origanum* genus in particular, have been shown to have strong anti-tumorigenic properties. For example, *O. vulgare* inhibits cell proliferation and induces apoptosis in human colon, stomach, hepatocarcinoma, and BC cell lines (Lombrea et al., 2020); *O. compactum* attenuates the proliferation of breast, lung and hepatoma cancer cell lines (Bouyahya et al., 2020); and *O. majorana* inhibits tumor growth and metastasis of numerous cancers including breast, colon, lung, pancreatic, lymphoblastic leukemia, and hepatocarcinoma (Bouyahya et al., 2021). Of particular interest, *O. syriacum* has been shown to exhibit an anti-proliferative effect on MCF-7 BC cells (Al-Kalaldeh et al., 2010; Husein et al., 2014) as well as human leukemia THP-1 cells (Ayesh et al., 2014). Furthermore, several phytochemicals present in *O. syriacum* have been reported to have anti-breast cancer potential. Some examples include naringenin, apigenin, carvacrol, thymoquinone, thymol, and rosmarinic which have been reported to impact the cancerous phenotype of BC cell lines (Kanno et al., 2005; Demain and Vaishnav, 2011; Lee et al., 2019; Messeha et al., 2020; Sampaio et al., 2021). In this study, LC-MS analysis of OSEE revealed the presence of vicenin-1, vicenin-2, orientin, isoorientin, vitexin, and isovitexin, all of which are flavonoid C-glycosides. Interestingly, vitexin, isovitexin, vicenin-1, and vicenin-2 are glycosides of apigenin (Engelhardt et al., 1993), which has anti-breast cancer activities and is reported to be present in *O. syriacum*, as discussed. In addition, these compounds were reported to have anti-cancerous activities, for example: vitexin against human leukaemia U937 cells, oesophageal cancer cells EC109; orientin against human cervical adenocarcinoma HeLa cells, oesophageal cancer EC109 cells, prostate cancer PC-3, DU-145 and LNCaP cells; isoorientin against liver hepatocellular carcinoma HepG2 cells; vicenin-2 agaisnt colorectal cancer HT29 cells, hepatocellular carcinoma HepG2, CA3, SNU-387, and HCCLM3 cells, prostate cancer cells PC-3, DU-145 and LNCaP (Nagaprashantha et al., 2011; Lee et al., 2012; Yuan et al., 2013; Guo et al., 2014; Yuan et al., 2014; Yang D et al., 2018; Huang et al., 2020). In the case of breast cancer, vitexin (Kim et al., 2018), orientin, and isoorientin (Czemplik et al., 2016) were reported to have anti-cancerous effects against MCF-7 breast cancer cells, which are not TNBC cells. Collectively, there are limited studies on the effect of these compounds in BC in general, and TNBC in particular, demanding investigation in future studies. Here, it is critical to mention that oftentimes, the whole herb or its crude extract may have more potent activity than a single or a combination of its bioactive constituents. This may partly be due to inherent synergistic effects between the various bioactive constituents, which could be lost when one or several bioactives are separately used (Rasoanaivo et al., 2011; Caesar and Cech, 2019; Zhao et al., 2020). The mechanism of this synergy has not been elucidated, yet several mechanisms may be operating in parallel (Caesar and Cech, 2019). For example, synergy could be due to the low bioavailability and poor pharmacokinetics of the individual bioactives, while the combination of bioactives may impart enhanced bioavailability (Caesar and Cech, 2019; Zhao et al., 2020). Future studies should test the anti-cancerous activities of single or combined bioactives of OSEE versus the activity of the whole extract. Overall, our results show that OSEE as a crude extract has potent *in vitro* anti-breast cancer activities and highlight OSEE as a potential source of natural compound(s) with anti-cancerous activities. Notwithstanding, further *in vivo* studies are needed to validate OSEE efficacy and safety in the treatment of TNBC.

We demonstrated that OSEE dose-dependently inhibited MDA-MB-231 proliferation. OSEE reduced the levels of the proliferation marker Ki67, which is highly expressed in TNBC and is associated with its aggressive pathologic features and poor clinical outcomes (Yang C et al., 2018). Reducing the levels of Ki67 further confirmed the potential of OSEE as a source of future TNBC therapeutics. We also showed that OSEE arrested MDA-MB-231 at the G_0_/G_1_ phase of the cell cycle. We investigated the potential correlation of the p38 MAPK (mitogen-activated protein kinase) pathway in OSEE-induced inhibition of MDA-MB-231 cell proliferation with cell cycle arrest. p38 signaling has been widely associated with anti-proliferative functions by regulating cell cycle progression and inducing apoptosis to maintain cellular homeostasis (Lafarga et al., 2009; Martínez-Limón et al., 2020). In addition, we showed that the levels of a downstream effector of p38, the cell cycle inhibitor protein p21 (Lafarga et al., 2009), increased remarkably with OSEE treatment. Overall, we showed that OSEE can inhibit proliferation of MDA-MB-231 cells and induce cell cycle arrest of TNBC cells at G_0_/G_1_ in a pathway that may involve p38/p21 signaling and downregulation of Ki67.

Reactive oxygen species are free radicals that occur in the body as by-products of mitochondrial and peroxisomal metabolism or the activity of certain enzymes like NADPH-oxidases (NOXs). ROS, at low concentrations, mediate important physiological processes, but an uncontrolled increase in their levels may precipitate pathological states. Therefore, it is critical to keep their levels under homeostatic control to avoid cellular oxidative stress resulting from an imbalance between the production of ROS and other reactive species and their elimination by cellular antioxidant systems (Posadino et al., 2019). Importantly, chronic slight elevations of ROS levels can cause cellular damage that may lead to carcinogenesis (Moloney and Cotter, 2018; Slika et al., 2022). However, this is not always the case as ROS was reported to have both pro- and anti-cancerous effects (Chio and Tuveson, 2017; Moloney and Cotter, 2018), dependent on ROS concentrations in the cell (Kong et al., 2000; De Sá Junior et al., 2017). Indeed, when ROS levels rise beyond a certain cytotoxic threshold, for example through exogenous application, they can cause the selective death of cancer cells mainly by apoptosis (Kong et al., 2000; De Sá Junior et al., 2017; Raza et al., 2017). Building on these results, targeting ROS signaling and inducing oxidative stress and/or inhibiting antioxidant processes has been envisaged as a mechanism for anticancer therapy (Chio and Tuveson, 2017; Kim et al., 2019). Nevertheless, the role of ROS in cancer therapy is full of intricacies and there are disputes over whether pro-oxidative or anti-oxidative therapies will be the effective course for the management of cancer (De Sá Junior et al., 2017). To complicate the situation even more, natural antioxidants themselves have been reported to either elevate or suppress ROS levels in cancer cells, and as a result, natural antioxidants have been reported to have either pro- or anti-cancerous effects depending on their concentration (Wang et al., 2021). Our results confirm this notion since OSEE exhibited high antioxidant potential *in vitro* by scavenging the DPPH radicals, but elevated ROS generation in MDA-MB-231 cells in culture. This was further manifested as a biphasic anti-proliferative effect mediated by OSEE in the presence of NAC as a ROS scavenger. Indeed, our results showed that only higher concentrations of OSEE were able to mitigate the inhibition of proliferation induced by the ROS, while lower concentrations of OSEE augmented this effect, indicating that the anti-proliferative effects of OSEE can be ROS-dependent in a biphasic manner. Biphasic concentration-dependent effects have been reported for many natural antioxidants (Shaito et al., 2020).

Cancer is a complex multifactorial disease mediated by multiple signaling pathways that regulate the expression of a wide array of genes implicated in tumor initiation, progression and metastasis, including the STAT3 signaling pathway. This pathway plays an important role in signaling associated with cellular proliferation, migration, invasion, and angiogenesis (Yuan et al., 2015). High levels of activated STAT3 have been observed in several types of cancer, including human BC (Levy and Inghirami, 2006). Activated STAT3 induces cancer transformation by affecting cellular pathways related to cell growth, apoptosis, and tumorigenesis. Interestingly, several studies have shown that STAT3 is constitutively activated in invasive BCs, but not benign tumors, indicating that STAT3 is mainly involved in tumor progression and metastasis, rather than tumor initiation (Watson and Miller, 1995; Ranger et al., 2009; Resemann et al., 2014). STAT3 has been attributed to have a key role in cell cycle regulation by mediating the progression of cells from G_1_ to S phase through the upregulation of D-cyclins and cell division cycle 25 A phosphatase (Cdc25A) and associated downregulation of cell cycle regulator proteins p21 and p27 (Leslie et al., 2006). In agreement with the roles of STAT3, our results showed that OSEE inhibited STAT3, activated p21, and induced G_0_/G_1_ cell cycle arrest. Moreover, ROS-mediated activation of p38 is reportedly implicated in cell cycle arrest at G_0_/G_1_ and induction of apoptosis in several cancers (Martínez-Limón et al., 2020). In this instance, p38 has been reported to activate p21, p27, or p57 (Martínez-Limón et al., 2020). Collectively, our findings suggest that OSEE exerts its anti-proliferative effects against BC by regulating several pathways, including those for STAT3, ROS, p38 MAP kinase, p21, and Ki67, satisfying the multi-target requirement of an effective anticancer therapeutic strategy, as already discussed.

STAT3 inhibits apoptosis by modulating key apoptosis regulators such as the pro-survival survivin (Gritsko et al., 2006), BCL-2 (Real et al., 2002), and p53 (Sp et al., 2021). As the “guardian of the genome,” p53 is critical in cell fate decisions in response to stress signals by inducing an arrest of the cell cycle, promoting DNA repair, and eliciting apoptosis. The activity of p53 is dependent on its quantity, integrity, and post-translational modification (Lacroix et al., 2006). For example, p53 phosphorylation at the N-terminal sites has been associated with increased protein stabilization and activity (Lacroix et al., 2006). Particularly, phosphorylation at the Ser15 site has been shown to be induced by almost all kinds of stress, disrupting the interaction of p53 with its major negative regulator MDM2 and increasing its binding to acetyltransferase P300 (Ito et al., 2001). Phosphorylation of mutant p53 at Ser15 restored its conformation to the wild-type form. Prospective therapies co-targeting STAT3 and p53 are sought to overcome cancer drug resistance (Pham et al., 2020). In this regard, several plant-derived compounds have been shown to exert their anticancer properties through inhibition of STAT3-signalling pathways. For example, the naturally occurring phytoalexin resveratrol inhibits the growth, progression and metastasis of BC cells by directly affecting STAT3 and its upstream regulators (Kohandel et al., 2021). Curcumin was also shown to suppress STAT3-signaling pathways and inhibit the growth of several cancers including breast, prostate, and pancreatic cancers (Hutzen et al., 2009; Lin et al., 2009; Liu et al., 2018). Here we reported similar results where OSEE induced apoptosis and downregulated the expression of STAT3. OSEE also induced the phosphorylation of p53, further supporting its multi-target properties. Phosphorylation of p53 can be associated with OSEE anticancer properties by stabilizing p53 to restore/increase its transcriptional activities. Moreover, OSEE treatment induced an accumulation of cells in the sub-G_0_ phase, activation of caspase-3, and downregulation of BCL-2, indicating that OSEE potentially induces p53-dependent intrinsic apoptosis in TNBC. In total, our data indicate that OSEE-induced apoptosis of MDA-MB-231 may be executed through a pathway involving STAT3, p53, p21, caspase-3, and BCL-2.

Epithelial–mesenchymal transition (EMT) is a hallmark of cancer progression to metastasis and involves loss of cell-cell adhesion and cell-extracellular matrix (ECM) linkages, crucial steps of metastasis (Singh and Settleman, 2010), and the association of TNBC with EMT is well documented (Jang et al., 2015; Jalaleddine et al., 2019). In our study, OSEE increased the formation of cell-cell aggregates, indicating enhanced cell-cell adhesion of MDA-MB-231 cells. This was concomitant with an increase in E-cadherin levels, suggesting that OSEE interferes with tumor growth and dissemination by acting on single migratory tumor cells rather than on migration of cell-clusters. Furthermore, MDA-MB-231 cells have reduced cellular adhesion and low levels of E-cadherin, having undergone extensive EMT (Jalaleddine et al., 2019). Increased levels of E-cadherin and cellular adhesion by OSEE treatment of MDA-MB-231 cells may indicate that OSEE has reversed EMT in these cells. Given that drug resistance is correlated with the extent of EMT (Du and Shim, 2016), it may be speculated that OSEE can reverse chemoresistance.

Patients with metastatic TNBC have a poor prognosis (Kassam et al., 2009). This is related to the robust invasive and migratory abilities of TNBC cells (Chang et al., 2013). The enhancement of cell migration and invasion is an essential part of the multistep process of cancer metastasis. It involves the dysregulation of cell-cell junctions and cell adhesion, and the degradation of ECM by proteases such as metalloproteinases (MMPs) (Mcgowan and Duffy, 2008; Dufour et al., 2011). In our study, wound-healing assays and trans-well migration and invasion assays were conducted to confirm the anti-migratory and anti-invasive properties of OSEE. Indeed, OSEE inhibited TNBC cell migration and invasion. Similarly, it has been demonstrated that *O. majorana,* a close relative of *O. syriacum*, also inhibited migration and invasion of TNBC cells through a mechanism involving the inhibition of MMP-2 and MMP-9 (Al Dhaheri et al., 2013). The expression of MMPs has been shown to be regulated by STAT3-mediated signaling processes (Alsamri et al., 2019), inviting a future investigation of the ability of OSEE to inhibit the invasive potential of MDA-MB231 through its action on STAT3/MMPs. Moreover, we showed that OSEE inhibited activation of FAK, which is normally overexpressed in cancer cells, and its high level correlates with the invasiveness and metastasis of human cancer (Weiner et al., 1993; Taliaferro-Smith et al., 2015; Choi et al., 2016). Whether OSEE-induced inhibition of migration and invasiveness of TNBC cells takes place solely through FAK-signaling or whether other routes are also involved remains to be tested in future studies. Overall, by enhancing cell adhesion and attenuating cell migration and invasion, OSEE may potently inhibit TNBC metastasis.

Tumors upregulate vascularization through angiogenesis in order to acquire nutrients and grow, and for later metastasis. Angiogenesis is associated with cancer invasion and metastasis (Brem et al., 1977; Brem et al., 1978). Particularly, TNBC has been associated with high microvascular density and consequently poor prognosis. Therefore, innovative cancer therapies have been directed to anti-angiogenic therapies to block tumor growth and metastasis (Braicu et al., 2016). This involves targeting pro-angiogenic factors such as inducible nitric oxide synthase (iNOS), which regulates the production of nitric oxide in response to external stimuli. Indeed, the expression of iNOS has been shown to be associated with microvascular density and to serve as a marker for the clinical staging of metastasis in gastric carcinoma (Song et al., 2002) and TNBC (Firger, 2015). Inhibition of iNOS was successful in decreasing TNBC aggressiveness, metastasis to the lungs in particular (Firger, 2015; Granados-Principal et al., 2015). Here, we showed that OSEE exhibits significant anti-angiogenic potential by reducing the formation of capillaries (decreased vessel length and junction number) on the chicken-egg CAM. This effect was correlated with a decrease in the levels of iNOS, suggesting that OSEE blocks angiogenesis through iNOS-mediated signaling pathways. Vascular endothelial growth factor (VEGF) is the predominant angiogenic factor of invasive human BC cells (Relf et al., 1997). VEGF expression is elevated in TNBC patients and is associated with poor prognosis (Linderholm et al., 2009). Extracts from *O. majorana* inhibit angiogenesis by downregulation of VEGF secretion (Al Dhaheri et al., 2013)*.* It is possible that OSEE-induced inhibition of angiogenesis takes place through a similar mechanism, in addition to downregulation of iNOS. The anti-angiogenic potential of *O. syriacum* further cements its potential use to develop anti-TNBC therapeutics.

## 5 Conclusion

In summary, our findings demonstrate that the ethanolic extract of *O. syriacum* (OSEE) exhibits potent anti-tumor growth and anti-metastatic effects on the aggressive phenotype of TNBC by modulating the processes of cell adhesion, migration, invasion, and angiogenesis through the inhibition of STAT3 signaling and activation of p38 MAPK signaling pathways. Moreover, OSEE caused cell cycle arrest, activated apoptosis, and inhibited angiogenesis in MDA-MB-231 cells. Therefore, due to its ability to modulate multiple pathways, OSEE is a potential source of candidate therapeutic anti-cancer agents. This warrants future investigation of OSEE as a source of novel compounds that can be used for multi-targeting of TNBC.

## Data Availability

The original contributions presented in the study are included in the article/Supplementary Material, further inquiries can be directed to the corresponding authors.
